# Role of FoxO1 and apoptosis in pulmonary vascular remolding in a rat model of chronic thromboembolic pulmonary hypertension

**DOI:** 10.1038/s41598-017-02007-5

**Published:** 2017-05-23

**Authors:** Chaosheng Deng, Zhanghua Zhong, Dawen Wu, Yunfei Chen, Ningfang Lian, Haibo Ding, Qiaoxian Zhang, Qichang Lin, Shuang Wu

**Affiliations:** Division of Respiratory and Critical Care Medicine, First Affiliated Hospital of Fujian Medical University, Fuzhou, Fujian Province 350005 China

## Abstract

To explore the role of FoxO1 and apoptosis in a rat model of chronic thromboembolic pulmonary hypertension (CTEPH). Rats were randomly divided into a sham group (n = 45) and an experimental group (n = 45). Autologous blood clots were injected into rats three times to induce CTEPH. Rats were further divided into three subgroups: a 1-week subgroup (n = 15), a 2-week subgroup (n = 15), and a 4-week subgroup (n = 15). Mean pulmonary arterial pressure (mPAP) and histopathology were evaluated at each time point. FoxO1, Bad, and Bcl-2 levels were examined at each time point using reverse transcription PCR and western blotting. The mPAP and vessel wall area/total area (WA/TA) ratio in the experimental group gradually increased in a time-dependent manner (*P* < 0.05). Both the mRNA and protein levels of FoxO1 decreased in the CTEPH rats compared to in the sham group. In addition, embolization led to the up-regulation of Bad and the down-regulation of Bcl-2 (*P* < *0.05*). FoxO1 and apoptosis play an important role in the pathogenesis of CTEPH. Apoptosis-resistant pulmonary artery endothelial cells may play an important role in remodeling of the rat pulmonary artery.

## Introduction

Chronic thromboembolic pulmonary hypertension (CTEPH) is a subtype of pulmonary hypertension (PH) (group 4)^1^. CTEPH is a sequela of acute pulmonary embolism (APE) induced by *in situ* thrombosis in the pulmonary artery^2^. It is characterized by vascular remodeling, pulmonary hypertension, and elevated pulmonary vascular resistance^3^. The risk factors for CTEPH include recurrent pulmonary embolism, large thrombi, and young age^4, 5^. Both pulmonary arterial endothelial cell (PAEC) and thrombosis lead to pulmonary vascular endothelial hyperplasia, thickening of the middle layer, and blood vessel occlusion^6–8^, however, the underlying mechanisms behind these processes have not been elucidated.

Apoptosis, also referred to as programmed cell death, is controlled by changes in the balance between pro-apoptotic (e.g., Bad, Bax, and p53) and anti-apoptotic (e.g., Bcl-2, Bcl-XL, and Bcl-AL) proteins. Inhibition of apoptosis can lead to the proliferation of cells in a manner similar to the proliferation seen in cancer cells.

Apoptosis has been shown to play an important role in pulmonary thromboembolism and ischemia/reperfusion injury^9^. In one study, Fan *et al*. showed that apoptosis regulates pulmonary vascular remolding in hypoxia-induced pulmonary hypertension^10^. Another study showed that increased proliferation of apoptosis-resistant PAECs occurs in idiopathic pulmonary hypertension^11^; moreover, these PAECs present had elevated factor VIII and Bcl-XL levels^12, 13^. However, it is unclear whether these changes occur in the vascular remolding process during the transition from pulmonary embolism to CTEPH. Forkhead box class O transcription factor 1 (FoxO1), a member of the FoxO family, plays critical roles in cell cycle, proliferation, apoptosis, and tumorigenesis by regulating expression of its target genes^14^. Based on this, we speculate that FoxO1 may play a direct role in PAEC apoptosis in CTEPH.

## Results

### Pulmonary arterial pressure in the rat model of CTEPH

**Figure 1:**
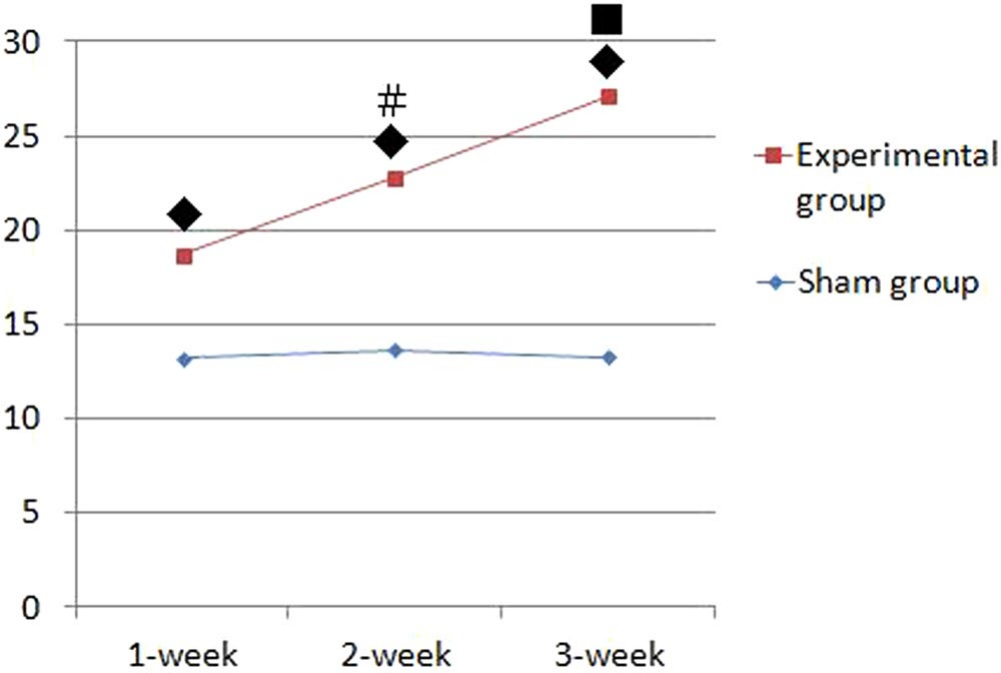
Pulmonary arterial pressure trace in the rat CTEPH model (mmHg). Note: Data are expressed as (mean ± SD)mmHg. ^◆^ *P* < 0.05, compared to the same week in the sham group; ^#^ *P* < 0.05, compared to the 1-week experimental subgroup; ^■^ *P* < 0.05, compared to the 1- and 2-week experimental subgroups.

As shown in Fig. 1, after repeated embolization, the mean pulmonary arterial pressure (mPAP) gradually increased over 4 weeks (27.11 ± 1.92 mmHg), with both the 2-week (22.81 ± 3.66 mmHg) and 4-week subgroups being significantly different than the 1-week subgroup (18.68 ± 1.73 mmHg) (*P* < 0.05). In contrast, there were no significant changes in mPAP values in the sham group over the same time period. At each individual time point, the experimental mPAPS were all significantly different from their respective sham values (*P* < 0.05).

### Pathological changes in the pulmonary artery in the rat CTEPH model

**Figure 2:**
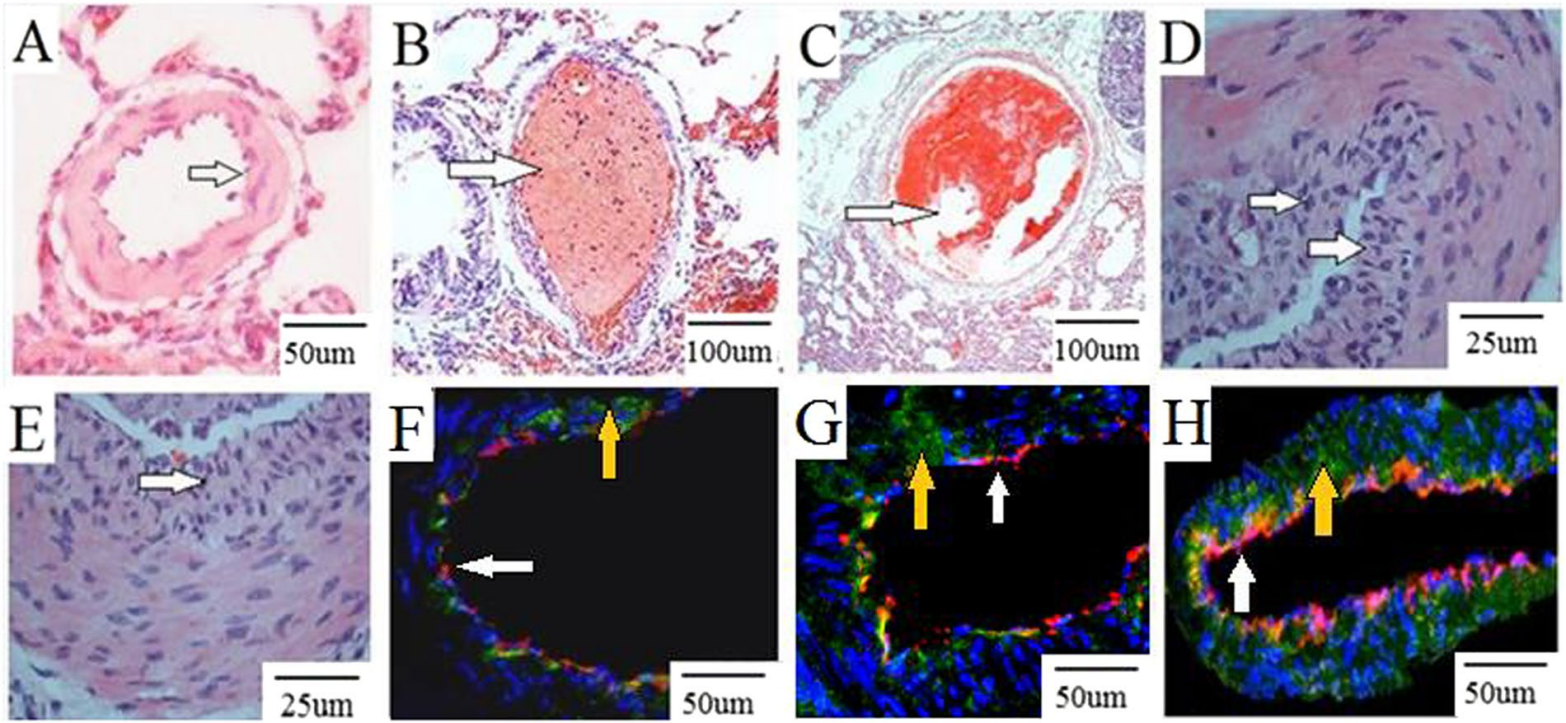
Pathological changes in the pulmonary artery in the rat CTEPH model. Note: No significant changes in the pulmonary artery were detected in the sham group (**A**). One hour after injection, pathological changes included connective tissue organization surrounding the reddish-brown thrombus (**B**); the thrombus (**C**) was smllar in the 1-week experimental subgroup. At 4-weeks, increased growth of pulmonary artery endothelial layer were detected (**D,E**). Vimentin and FVIII factor in sham group (**F**), 1-week (**G**) and 2-week (**H**) experimental subgroup. Red light: FVIII factor (white arrows), green light: vimentin (yellow arrows), blue light: nuclear.

As shown in Fig. 2, no significant changes in the pulmonary artery were detected in the sham group (A). One hour after injection, pathological changes included connective tissue organization surrounding the reddish-brown thrombus (B); the thrombus (C) was smllar in the 1-week experimental subgroup. At 4-weeks, we detected increased growth of pulmonary artery endothelial cells (D, E). Double fluorescence immune staining for FVIII factor (red light) and vimentin (green light) in pulmonary artery wall was shown in sham group (F) and higher expression in 1-week (G) and higher expression in 2-week (H) experimental subgroup. The percentage of muscularized vessel count was showed in Table 1.

**Table 1:** The percentage of muscularized vessel count in the rat CTEPH model.

Group	Percentage of muscularized vessel count
Sham group	0.00 ± 0.00
1-week subgroup	0.16 ± 0.09^◆^
2-week subgroup	0.26 ± 0.07^◆#^
4-week subgroup	0.63 ± 0.07^◆∗^

Note: Data are expressed as (mean ± SD) %. ^◆^ *P* < 0.05, compared to the sham group; ^#^ *P* < 0.05, compared to the 1-week experimental subgroup; ^∗^ *P* < 0.05, compared to the 1- and 2-week experimental subgroups.

As shown in Table 2, the wall area/total area (WA/TA) ratio gradually increased following repeated embolization in the 1-, 2-, and 4-week experimental subgroups, with both the 2-week and 4-week subgroups being significantly different from the 1-week subgroup. No changes in the WA/TA ratio were seen in the sham group over time. At each individual time point, the experimental WA/TA ratios were all significantly different from their respective sham values (*P* < 0.05).

**Table 2:** WA/TA ratio in the rat CTEPH model.

Group	Sham group	Experimental group
1-week subgroup	30.22 ± 5.65	44.47 ± 6.32^◆^
2-week subgroup	29.13 ± 6.76	48.45 ± 7.28^◆#^
4-week subgroup	29.65 ± 5.83	52.43 ± 9.32^◆∗^

Note: Data are expressed as (mean ± SD)%. ^◆^ *P* < 0.05, compared to the same week in the sham group; ^#^ *P* < 0.05, compared to the 1-week experimental subgroup; ∗*P* < 0.05, compared to the 1- and 2-week experimental subgroups.

### Endothelial cell apoptosis detected by TUNEL in the rat CTEPH model

**Figure 3:**
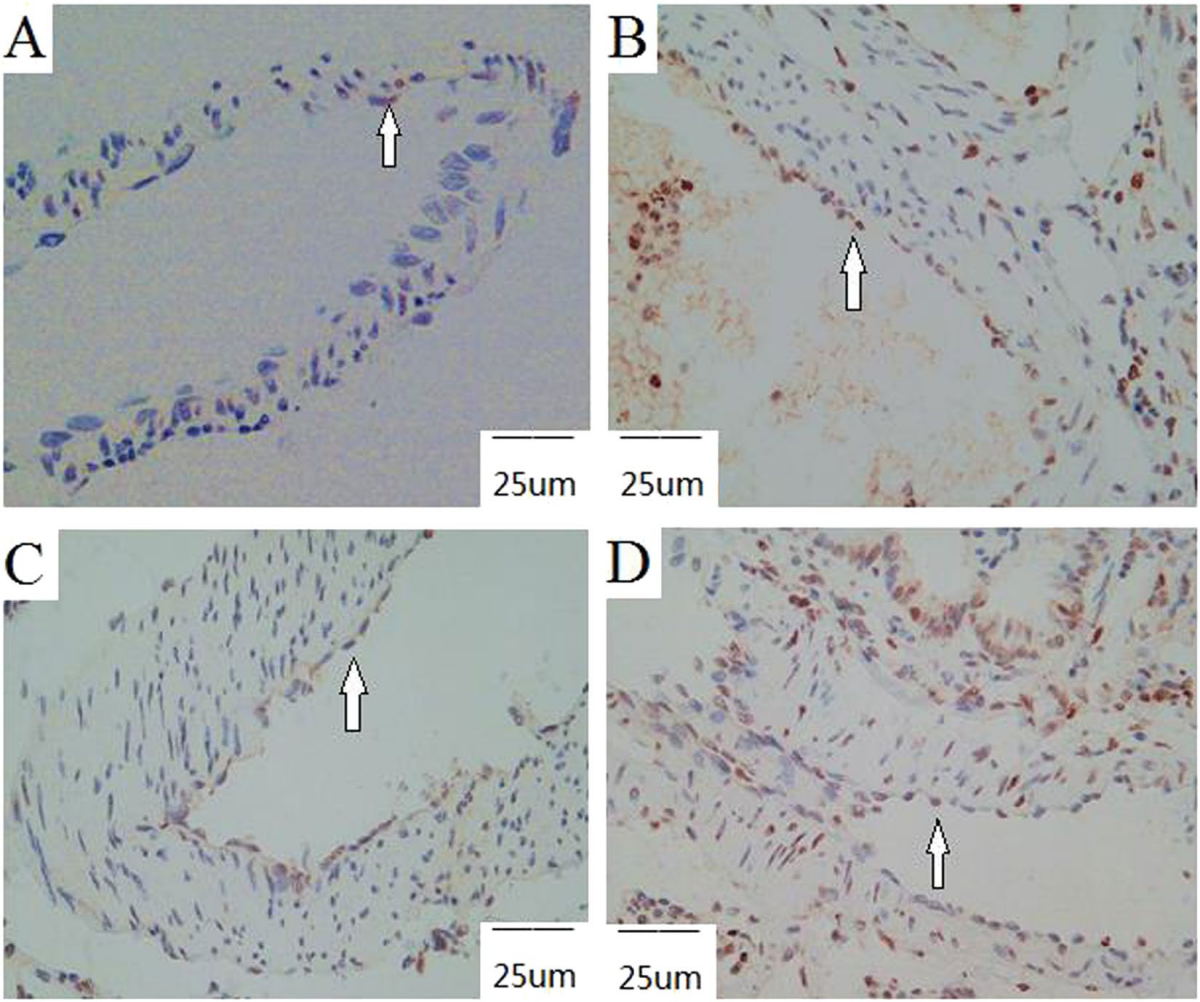
Endothelial cell apoptosis detected by TUNEL in the rat CTEPH model. Note: Apoptotic cells (white arrows). The endothelial cell apoptotic activity of the sham group was the lowest (**A**). In the experimental group, these cells’ apoptotic activity decreased with the prolongation of time (**B–D**).

Endothelial cell apoptosis detected by TUNEL gradually decreased in the 1(B), 2(C) and 4(D) week experimental subgroups, and were higher than the sham group (A) (Fig. 3). Endothelial cell apoptosis index was shown in Table 3.

**Table 3:** Endothelial cell apoptosis index in the rat CTEPH model.

Group	Sham group	Experimental group
1-week subgroup	9.59 ± 0.42	35.80 ± 3.49^◆^
2-week subgroup	9.82 ± 0.45	22.39 ± 2.02^◆#^
4-week subgroup	10.16 ± 0.68	16.07 ± 2.02^◆∗^

Note: Data are expressed as (mean ± SD)%. ^◆^ *P* < 0.05, compared to the same week in the sham group; ^#^ *P* < 0.05, compared to the 1-week experimental subgroup; ^∗^ *P* < 0.05, compared to the 1- and 2-week experimental subgroups.

### mRNA levels of FoxO1, Bad, and Bcl-2 in the rat CTEPH model

**Figure 4:**
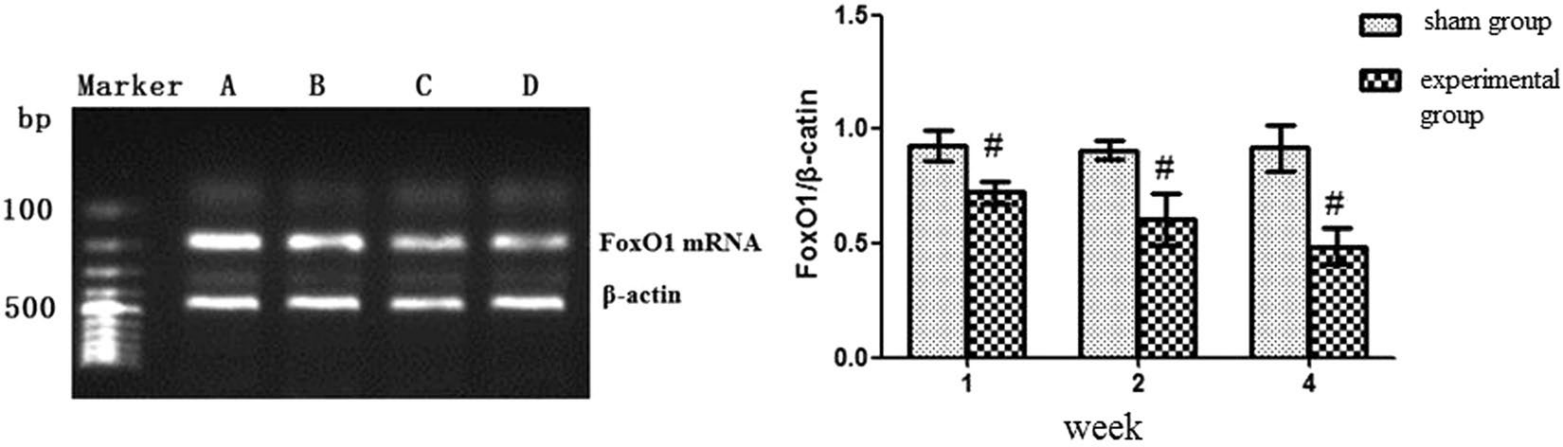
FoxO1 mRNA expression in the rat CTEPH model. Note: (**A**) Sham group; (**B**) 1-week experimental subgroup; (**C**) 2-week experimental subgroup (**D**) 4-week experimental subgroup. ^#^ *P* < *0.05*, compared to sham group within the same subgroup.

As shown in Fig. 4, the mRNA expression of FoxO1 gradually decreased in the 1-(0.72 ± 0.05), 2-(0.60 ± 0.11), and 4-(0.48 ± 0.08) week experimental subgroups. In contrast, no changes in FoxO1 mRNA expression occurred in the 1-(0.92 ± 0.07), 2-(0.90 ± 0.04), and 4-(0.91 ± 0.10) week sham subgroups. At each time point, the FoxO1 levels in the experimental group were lower than sham group, and FoxO1 levels in the experimental subgroups were significant different from those in the sham subgroups (*P* < 0.05).

**Figure 5:**
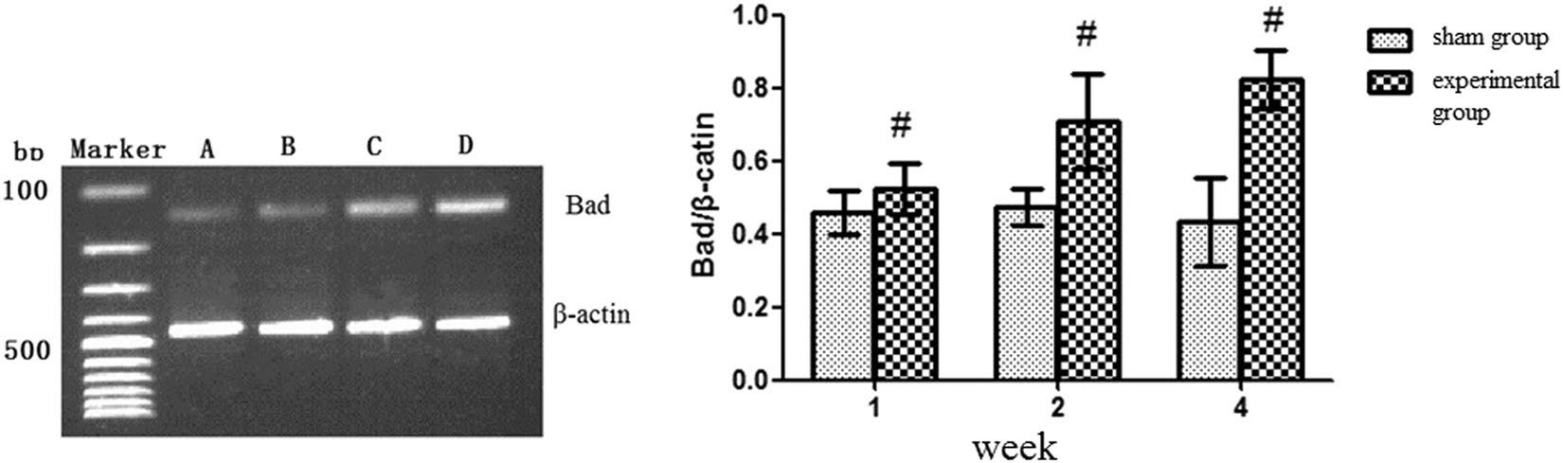
Bad mRNA expression in the rat CTEPH model. Note: (**A**) Sham group; (**B**) 1-week experimental subgroup; (**C**) 2-week experimental subgroup; (**D**) 4-week experimental subgroup. ^#^ *P* < *0.05*, compared to sham group within the same subgroup.

As shown in Fig. 5, the mRNA expression of Bad gradually increased in the 1-(0.52 ± 0.07), 2-(0.70 ± 0.13), and 4-(0.82 ± 0.08) week experimental subgroups. In contrast, no changes in Bad mRNA expression occurred in the 1-(0.45 ± 0.06), 2-(0.47 ± 0.05), and 4-(0.43 ± 0.12) week sham subgroups. At each time point, the Bad levels in the experimental group were higher than sham group, and Bad levels in the experimental subgroups were significant different from those in the sham subgroups (*P* < 0.05).

**Figure 6:**
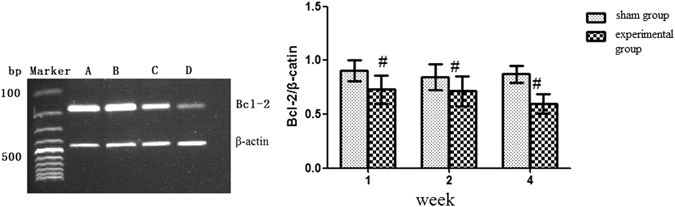
Bcl-2 mRNA expression in the rat CTEPH model. Note: (**A**) Sham group; (**B**) 1-week experimental subgroup; (**C**) 2-week experimental subgroup; (**D**) 4-week experimental subgroup. ^#^ *P* < *0.05*, compared to sham group within the same subgroup.

As shown in Fig. 6, the mRNA expression of Bcl-2 remained stable in the 1-(0.72 ± 0.13), and 2-(0.71 ± 0.14) week experimental subgroups, and then decreased in the 4-(0.59 ± 0.09) week experimental subgroup. In contrast, no changes in Bcl-2 expression occurred in the 1-(0.90 ± 0.10), 2-(0.84 ± 0.12), and 4-(0.87 ± 0.08) week sham subgroups. At each time point, the Bcl-2 levels in the experimental group were lower than sham group, and Bcl-2 levels in the experimental subgroups were significant different from those in the sham subgroups (*P* < 0.05). Bcl-2 expression was not significantly different between the 1-week and 2-week experimental subgroups (*P > *0.05) whereas the Bcl-2 mRNA expression was significantly reduced in the 4-week subgroup compared to in the 1-week and 2-week experimental subgroups (*P* < 0.05).

### Protein levels of FoxO1, Bad, and Bcl-2 in the rat CTEPH model

**Figure 7:**
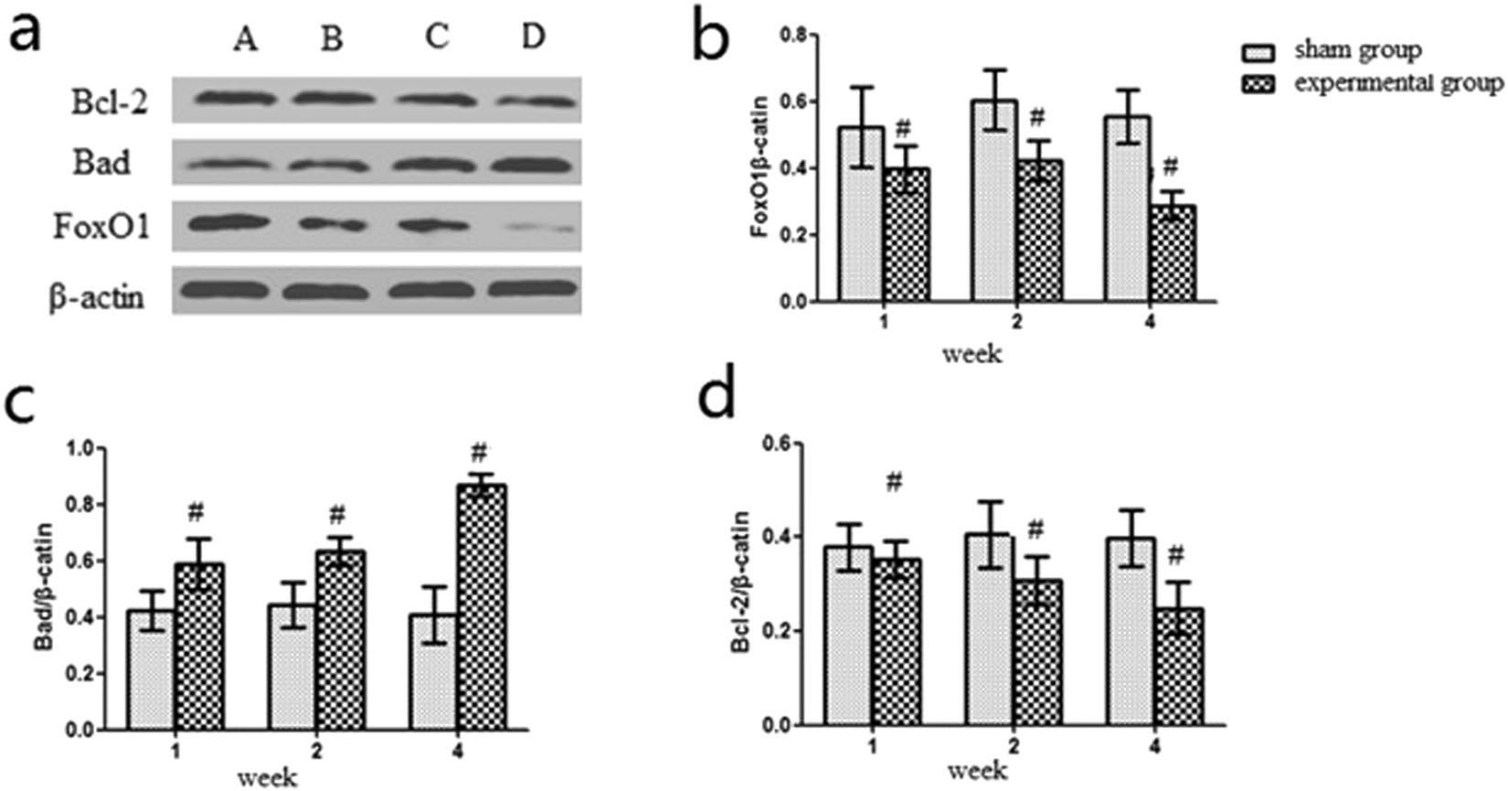
FoxO1, Bad, and Bcl-2 protein expression in the rat CTEPH model. Note: (**a**) Sham group; (**b**) 1-week experimental subgroup; (**c**) 2-week experimental subgroup; (**d**) 4-week experimental subgroup. ^#^ *P* < *0.05*, compared to sham group within the same subgroup. *P < *0.05*, compared to 1- and 2-week experimental subgroups.

Protein expression of FoxO1, Bad, and Bcl-2 in this model was shown in Fig. 7a. As shown in Fig. 7b, the protein expression of FoxO1 was lower in the 1-, 2-, and 4- week experimental subgroups compared to the same week sham subgroups, respectively (*P* < 0.05). The protein expression of FoxO1 decreased in the 4-week experimental subgroup compared to in the 1- and 2-week experimental subgroups (*P* < 0.05) (Table 4). The protein expression of Bad was higher in the 1-, 2-, and 4-week experimental subgroups compared to the same week sham subgroups, respectively (*P* < 0.05). Bad expression was increased in the 4-week experimental subgroup compared to in the 1- and 2-week experimental subgroups (*P* < 0.05) (Fig. 7c). Repeated embolization inhibited the expression of Bcl-2 in the 1-, 2-and 4-week experimental subgroups compared to the same week sham subgroups (*P* < 0.05) (Fig. 7d).

**Table 4:** FoxO1, Bad, and Bcl-2 protein expression in the rat CTEPH model.

Group	FoxO1	Bad	Bcl-2
1-week sham group	0.52 ± 0.12	0.42 ± 0.07	0.37 ± 0.05
2-week sham group	0.60 ± 0.09	0.44 ± 0.08	0.38 ± 0.11
4-week sham group	0.55 ± 0.08	0.40 ± 0.10	0.36 ± 0.08
1-week subgroup	0.39 ± 0.07^#^	0.58 ± 0.09^#^	0.35 ± 0.03^#^
2-week subgroup	0.42 ± 0.06^#^	0.63 ± 0.05^#^	0.30 ± 0.04^#^
4-week subgroup	0.28 ± 0.04^#*^	0.86 ± 0.04^#*^	0.24 ± 0.06^#*^

Note: ^#^ *P* < *0.05*, compared to sham group within the same subgroup. *P < *0.05*, compared to 1- and 2-week experimental subgroups.

### Immunohistochemical staining of FoxO1, Bad, and Bcl-2 in the rat CTEPH model

**Figure 8:**
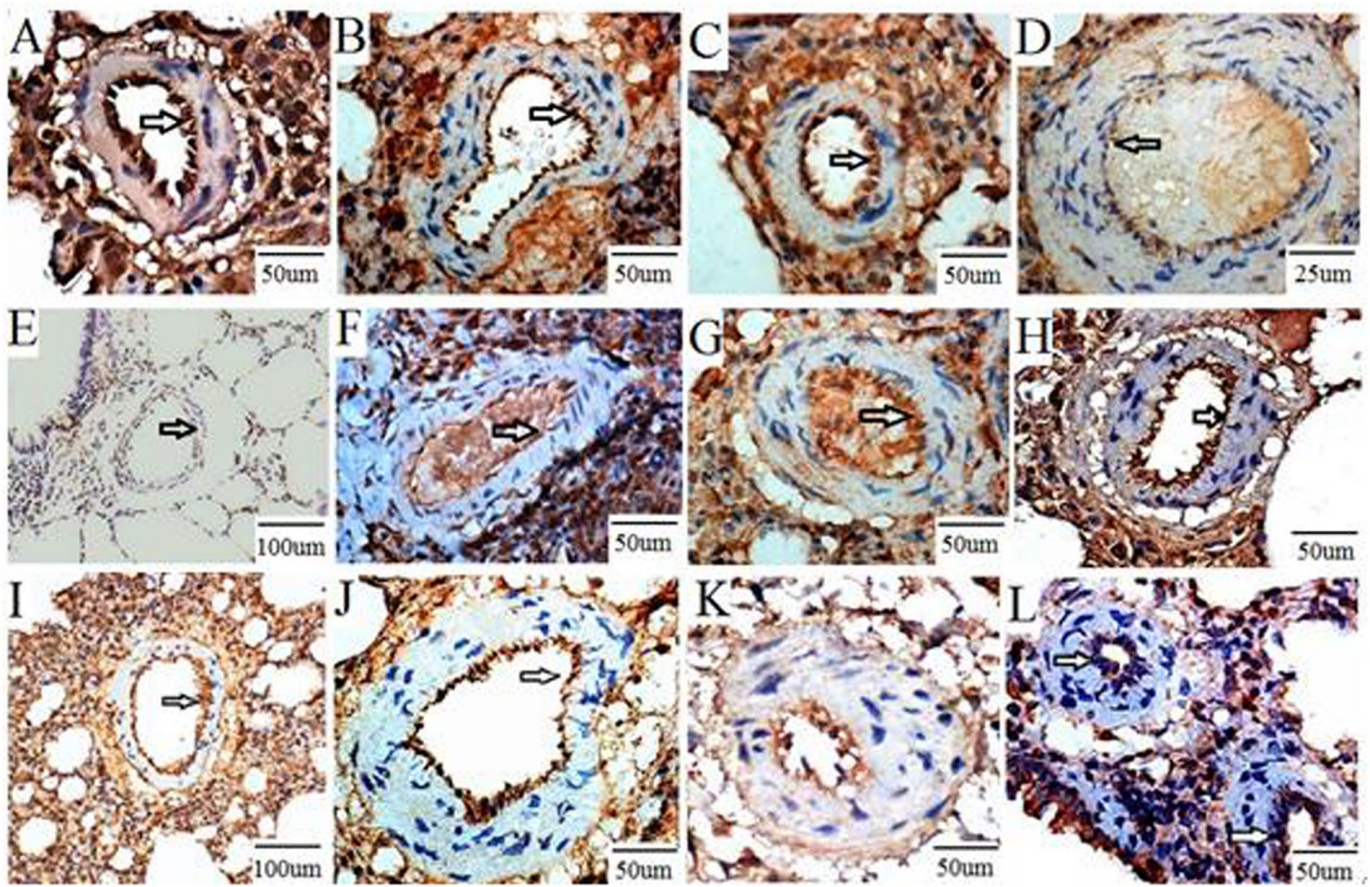
Immunohistochemical staining of FoxO1, Bad, and Bcl-2 in the rat CTEPH model. Note: FoxO1 expression, both nuclear and cytoplasmic, was ‘strong positive’ in all sham subgroups (**A**), ‘positive’ in the 1-week (**B**) and 2-week (**C**) experimental subgroups, and ‘weak positive’ in the 4-week experimental subgroup (**D**). Bad expression, both nuclear and cytoplasmic, was ‘negative’ in all sham subgroups (**E**), ‘positive’ in both the 1-week (**F**) and 2-week (**G**) experimental subgroups, and ‘strong positive’ in the 4-week experimental subgroup (**H**). Bcl-2 expression, both nuclear and cytoplasmic, was ‘positive’ in all sham subgroups (**I**), ‘strong positive’ in the 1-week experimental subgroup (**J**), ‘positive’ in the 2-week experimental subgroup (**K**), and ‘weak positive’ in the 4-week experimental subgroup (**L**).

As shown in Fig. 8, an immunohistochemical analysis of FoxO1, Bad, and Bcl-2 expression in the pulmonary artery endothelial cells was conducted. FoxO1 expression, both nuclear and cytoplasmic, was ‘strong positive’ in all sham subgroups (A), ‘positive’ in the 1-week (B) and 2-week (C) experimental subgroups, and ‘weak positive’ in the 4-week experimental subgroup (D). Bad expression, both nuclear and cytoplasmic, was ‘negative’ in all sham subgroups (E), ‘positive’ in both the 1-week (F) and 2-week (G) experimental subgroups, and ‘strong positive’ in the 4-week experimental subgroup (H). Bcl-2 expression, both nuclear and cytoplasmic, was ‘positive’ in all sham subgroups (I), ‘strong positive’ in the 1-week experimental subgroup (J), ‘positive’ in the 2-week experimental subgroup (K), and ‘weak positive’ in the 4-week experimental subgroup (L).

### Pearson correlation coefficients for mPAP, WA/TA, FoxO1, Bad, and Bcl-2

As shown in Table 5, in the experimental group, mPAP had a positive correlation with the WA/TA ratio (*r* = 0.589, *P* < 0.05). FoxO1 protein expression had a negative correlation with WA/TA (*r* = −0.445, *P* < 0.05), mPAP (*r* = −0.510, *P* < 0.05), and Bad protein levels (*r* = −0.486, *P* < 0.05); and a positive correlation with Bcl-2 protein expression (*r* = 0.535, *P* < 0.05). Bad protein levels were positively correlated with WA/TA (*r* = 0.645, *P* < 0.05) and mPAP (*r* = 0.570, *P* < 0.05). Bcl-2 protein levels had a negative correlation with WA/TA (*r* = −0.716, *P* < 0.05) and mPAP (*r *= −0.608, *P* < 0.05).

**Table 5:** Pearson correlation coefficients for mPAP, WA/TA, FoxO1, Bad, and Bcl-2.

	WA/TA	mPAP	FoxO1
mPAP	0.589 (*p* = 0.005)	—	—
FoxO1	−0.445 (*p* = 0.002)	−0.510 (*p* = 0.001)	—
Bad	0.645 (*p* = 0.000)	0.570 (*p* = 0.010)	−0.486 (*p* = 0.016)
Bcl-2	−0.716 (*p* = 0.002)	−0.608 (*p* = 0.000)	0.535 (*p* = 0.001)

## Discussion

### Apoptosis and vascular remolding in CTEPH

PH is a complex disease that is regulated by a variety of pathogenic factors or gene mutations. In PH, persistent vasoconstriction and vascular remolding are the two most common pathological changes. Increased proliferation of endothelial-like cells (mesenchymal cells) along with smooth muscle cell migration into the intima can lead to the thickening of the pulmonary artery; smooth muscle can also be found in the distal capillary^6, 15, 16^. Structural and functional defects within endothelial-like cell mitochondria promote thrombosis^17^. Inflammation induced by a pulmonary embolism enhances the interaction between PAEC injury and thrombosis, further aggravating the vascular injury and promoting remolding; the latter is characterized by intimal thickening, increased pulmonary vascular resistance, and pulmonary hypertension^13^. Pulmonary vascular remolding, the pathological basis of CTEPH, is characterized by endothelial injury, proliferative expansion of smooth muscle cells, and blood vessel occlusion^8, 18^. In order to understand CTEPH, we established a rat model using repeated injection of autologous clots along with TXA treatment. The inflammation, PAEC injury, and thrombosis following the induction of a pulmonary embolism may contribute to the pathogenesis of pulmonary vascular remolding. In our study, both mPAP and WA/TA gradually increased at 1, 2, and 4 weeks following induction of a pulmonary embolism. In addition, mPAP was positively correlated with WA/TA, indicating that the arterial pressure elevation was related to the vascular remolding that occurred following the pulmonary embolism.

Pulmonary vasculopathy usually leads to malignant features in the vasculature, such as hyper-proliferation, apoptosis-resistance, monoclone, and gene mutations^19, 20^. Cell growth and apoptosis are in a dynamic balance, and a disturbance in this balance leads to cellular dysfunction. Various stimuli (e.g., injury and hypoxia) can lead to structural and functional changes in the pulmonary artery wall, which subsequently produces an imbalance between cell survival and apoptosis, thereby triggering pulmonary vascular remolding^21^. Lee *et al*. have shown that intimal proliferation plays an important role in the formation of clustered lesions of arterioles in idiopathic pulmonary hypertension^22^. Levy *et al*. have found that smooth muscle cells participate in vascular remolding, and that the anti-apoptotic protein Bcl-2 is either not expressed, or is expressed at very low levels, in smooth muscle cells; consistent with our study, their data suggest that Bcl-2 is highly expressed in pulmonary endothelial cells, indicating that apoptosis-resistant endothelial cells play a critical role in pulmonary vascular remolding^23^. In our study, factor VIII, a specific marker for endothelial cells and vimentin, a protein expressed in stromal cells and smooth muscle cells expressed in the pulmonary artery wall, as shown by double fluorescence immune staining confirmed the obvious pulmonary artery remolding, especially pulmonary artery endothelial layer with the time prolongation after APE. Apoptosis of endothelial cells in experimental group suggested endothelial cells apoptosis-resistant.

Some researchers believe that pulmonary hypertension is a syndrome and the endothelial cell apoptosis plays a critical role. Several possible mechanisms have been proposed including the following: apoptosis induces the defluvium of endothelial cells and the collapse of pre-capillary arterioles, thereby enhancing pulmonary artery resistance; apoptotic endothelial cells lose their suppressive effect on smooth muscle cell proliferation, leading to pulmonary remolding; the apoptosis-resistant endothelial cells are enriched by the apoptotic process^24, 25^. In the present study, levels of the mRNA encoding the pro-apoptotic protein Bad gradually increased following repeated embolism. Immunohistochemistry showed that the pulmonary embolism increased Bad protein expression in endothelial cells. In parallel, the levels of the mRNA encoding the anti-apoptotic protein Bcl-2 decreased following pulmonary embolism induction. Following pulmonary embolism induction, the protein of Bad expression was positively correlated with both the WA/TA ratio and mPAP. In contrast, Bcl-2 was negatively correlated with the WA/TA ratio and mPAP. Taken together, these data suggest that apoptosis plays an important role in vascular remolding following pulmonary embolism. The precise mechanisms need to be further investigated.

### Expression of FoxO1 in pulmonary embolism-induced vascular remolding

The FoxO transcription factors play critical roles in cell proliferation, apoptosis, and differentiation by regulating the PI3K/Akt signaling pathway and its downstream genes (e.g., genes encoding Bcl-2/Bcl-XL, Bad, glycogen synthase kinase)^26–28^. FoxO1 is expressed in the embryonic vasculature and FoxO1 gene defects cause embryonic death, suggesting that FoxO1 is essential for maintenance of embryonic vasculature and normal physiological function. In the presence of vascular endothelial growth factor (VEGF), FoxO1-deficient endothelial cells exhibit clearly different morphological changes compared to the normal endothelial cells, indicating that FoxO1 is required for the normal physiological function of endothelial cells. In the present study, we found that pulmonary embolism decreased the mRNA and protein expression of FoxO1 compared to the sham group, possibly through a mechanism whereby downregulation of FoxO1 promotes proliferation of endothelial cells leading to vascular remodeling. In a study in umbilical veins, activation of the PI3K/Akt pathway in endothelial cells induces the deregulation of FoxO1 and subsequently inhibits the secretion of connective tissue growth factor (CTGF), leading to the abnormal proliferation of endothelial cells.

In our study, repeated embolization led to decreased expression of FoxO1 and Bcl-2, and increased Bad levels in the pulmonary artery. Pearson correlation coefficient analysis showed that FoxO1 had a negative correlation with WA/TA, mPAP, and Bad protein expression, and a positive correlation with Bcl-2 expression. To a certain extent, these data suggest that FoxO1 and apoptosis are implicated in pulmonary embolism-induced vascular remolding by upregulating Bad and downregulating Bcl-2. The immunofluorescent staining of factor VIII in endothelial cells suggested that apoptosis-resistant endothelial cells may induce vascular remolding following pulmonary embolism.

### Study limitations

Although a CTEPH model has been successfully established in rats in this study, the pulmonary endothelial cells were difficult to isolate and *in vitro* assays have not been performed. It is a key point to conduct a functional assay using isolated pulmonary artery endothelial cells from CTEPH rats and from patients undergoing PEA. Therefore, the mechanism for FoxO1 and apoptosis-induced pulmonary remolding in CTEPH have been left to be investigated further.

## Materials and Methods

### Animals

Ninety male Sprague Dawley (SD) rats (two months old, 250–300 g) were purchased from the Experimental Animal Center of Fujian Medical University (China). All animals were kept in an air-conditioned room at 20–24 °C and 65–70% relative humidity. All animals had free access to food and water. All experimental and animal care protocols were approved by the animal ethics committee of Fujian Medical University and guided for the Care and Use of Laboratory Animals (NIH, Bethesda, MD, USA).

### Grouping

Ninety SD rats were randomly sorted into a sham group (n = 45) and an experimental group (n = 45). In the experimental group, autologous blood clots were injected into the left external jugular vein and the same operation was repeated 4 and 7 days after the first injection. We prepared the blood clots one day ahead of the injection time, and we injected 32 ± 6 blood clots into 1 ml normal saline and injected mixed liquor at a speed of 0.2 ml/min. Meanwhile the shape of the blood clots is cylinder with 1 mm in diameter and 2 mm–3 mm in length. The procedures in the sham group were the same as in the experimental group except that rats were administered normal saline via the left jugular vein. The rats were further divided into three subgroups according to the time of observation: a 1-week subgroup (n = 15), a 2-week subgroup (n = 15), and a 4-week subgroup (n = 15).

### Autologous blood clots preparation

The rats were anesthetized with an intraperitoneal injection of 10% chloral hydrate (0.3 g/kg). Blood was collected from the orbital vein using a capillary glass tube (inner diameter, 1 mm) and placed in sterilized petri dishes overnight. Blood clots were washed with normal saline and trimmed to 3 mm in length. The clots were then aspirated into a syringe with 1.5 ml normal saline containing tranexamic acid (TXA) (200 mg/kg/rat) and connected to a catheter with a 7 F needle for later use.

### Animal model establishment

Rats were injected intraperitoneally with 10% chloral hydrate (0.3 g/kg) and placed on the operating table. The left external jugular vein was then injected with the prepared blood clots. Rats in the sham group were injected with an equal volume of normal saline also via the left jugular vein. The injection procedure was repeated 4 and 7 days after the first injection. Penicillin (10,000 U/kg/day) was injected for 3 days after the operation to prevent microbial infection, meanwhile endogenous fibrinolysis was inhibited with an intraperitoneal injection of tranexamic acid (TXA, 200 mg/kg, once/day).

### Sample collection

After anesthetization, the right external jugular vein was located and pulmonary arterial pressure was determined. Arterial blood (2 mL) was collected via the aorta abdominalis, and EDTA (100 g/L) was then added as an anti-coagulant. Blood was preserved at −80 °C after centrifugation at 3000 rpm at 4 °C for 15 min. The steps of dissection and fixation of the lung as follows: The rat thoracotomy was performed and the lungs were gently extracted out. The pulmonary artery were gently washed the blood through by normal saline before fixation Half of the a lung were fixed in 10% formalin and embedded in paraffin. These sections were then subject to hematoxylin and eosin (HE) staining, immunohistochemistry, and immunofluorescence. The other half were used to extracted the pulmonary arteries which were stored at −80 °C and used in reverse transcription PCR and western blotting.

### Pulmonary arterial pressure measurement

Rats were injected intraperitoneally with 10% chloral hydrate (0.3 g/kg) at either 1, 2, or 4 weeks. The right external jugular vein was located, and a PE-50 polyvinyl chloride (PVC) catheter connected to a pressure transducer and biological signal acquisition system was slowly inserted. The catheter position was determined by monitoring changes in the pressure curve waveform and pulmonary arterial pressure was recorded after the catheter was extended into the pulmonary artery via the right ventricle.

### Image analysis

Pathological changes in the pulmonary arteries were observed using an optical microscope (Leica DMI3000M, Germany) (X 400) and the pulmonary arterial (50–100 μm) vessel wall area/total area (WA/TA) ratio was calculated using an image processing system (Rencongzhong Co. Ltd., Xuzhou, China).

## Expression of FoxO1, Bad, and Bcl-2

### Immunohistochemistry

After rat pulmonary artery sections were deparaffinized and rehydrated, sections were incubated with rabbit anti-rat FoxO1, Bad, and Bcl-2 polyclonal antibodies (at 1:100, 1:150, 1:150, respectively; Abcam (Shanghai) trading co., LTD, Shanghai, China) according to previous study^15^. Sections were treated with the Biotin-Streptavidin Horseradish Peroxidase (HRP) Detection System (ZSGB-BIO, Shanghai, China). Immunoreactivity was visualized using 3,3′-diaminobenzidine (DAB). Immunofluorescence was used to measure the expression of factor VIII in the pulmonary artery. Double fluorescence immune staining was used to detect the FVIII and vimentin in the pulmonary artery. Pulmonary artery endothelial apoptosis was detected by apoptosis TUNEL detection kit.

### RT-PCR

RNA was extracted from the pulmonary artery tissue samples with the Trizol reagent (Invitrogen, Carlsbad, CA, USA) and the purity of the RNA was analyzed using spectrophotometer (MACY (China) INSTRUMENTS INC, Shanghai, China) according to previous study^15^. Primer pairs were designed for FoxO1, Bad, and Bcl-2, and β-actin (internal control) using their DNA sequences (Sangon Biotech Co., Ltd, Shanghai, China). FoxO1 forward: 5′-AATTTGCTAAGAGCCGAGGA-3′ and FoxO1 reverse: 5′-AAGTCATCATTGCTGTGGGA-3′; Bad forward 5′-AGA TGG AGG TGG AGA TGT GG-3′ and Bad reverse: 5′-AAC AGC AGG TCT TTC CCA AG-3′; Bcl-2 forward: 5′-CCTCTACGGCCCCTTGTCG-3′ and Bcl-2 reverse 5′-ATCCTCCCCCAGTTCAC CCCATCC-3′; β-actin forward: 5′-AAC CCT AAG GCC AAC CGT G-3;’ and β-actin reverse: 5′-TGC TCG AAG TCT AGG GCA AC-3′. Cycling conditions were as follows: 94 °C denaturation for 5 min; 35 cycles of 94 °C for 30 sec, 60 °C for 30 sec, and 72 °C for 30 sec; and a final 10 min 72 °C extension. FoxO1, Bad, and Bcl-2 expression was semi-quantitatively determined with the imaging software.

### Western blot analysis

FoxO1, Bad, and Bcl-2 protein expression was analyzed by western blot analysis according to previous study^15^. FoxO1, Bad, and Bcl-2 polyclonal antibodies were used to incubate the membranes (1:500, 1:500, 1:1000, respectively; Abcam (Shanghai) trading co., LTD, Shanghai, China). The targeted proteins were analyzed using the Lab-work image analysis software (Gene Company Limited, Hong Kong, China).

### Statistical analysis

Experimental data were analyzed by SPSS17.0 and are presented as mean ± standard deviation (SD). Differences between experimental groups were determined by variance analysis followed by an LDS or a Games-Howell test. Correlation between groups was determined using a Pearson correlation. P value < 0.05 was considered as significantly different.
